# *Vital Signs*: Progress Toward Eliminating HIV as a Global Public Health Threat Through Scale-Up of Antiretroviral Therapy and Health System Strengthening Supported by the U.S. President’s Emergency Plan for AIDS Relief — Worldwide, 2004–2022

**DOI:** 10.15585/mmwr.mm7212e1

**Published:** 2023-03-24

**Authors:** Helen M. Chun, Emilio Dirlikov, Mackenzie Hurlston Cox, Michelle Williams Sherlock, Yaa Obeng-Aduasare, Kimi Sato, Andrew C. Voetsch, Abraham D. Ater, Erin Rottinghaus Romano, Hank Tomlinson, Surbhi Modi, Angeli Achrekar, John Nkengasong, CDC Global HIV Working Group, CDC Global HIV Working Group, Simon Agolory, Josef Amann, Brittney Baack, Stephanie Behel, Anand Date, Jeff Hanson, William P. Killam, Hetal Patel, Sadhna Patel, Rituparna Pati, Laura Porter, Alicia Warner, Tadesse Wuhib, Clement Zeh, Ana Carolina Faria E Silva Santelli, Giselle Guevara, Rosa Elena Morales, Alexandre Kunumboa Ekra, Francois Kitenge, Luis Bonilla, Sikhathele Mazibuko, Tekeste Damena, Patrice Joseph, Sunita Upadhyaya, Indira Aitmagambetova, Jane Mwangi, Nazira Usmanova, Douangchanh Xaymounvong, Mugyenyi Asiimwe, Maida Alice, Gillian Jessina Masamha, Gram Mutandi, Solomon Odafe, Lacson Romel, Canisious Musoni, Mary Mogashoa, Alex Bolo, Aziz Nabidzhonov, George Mgomella, Rangsima Lolekha, Stella Alamo-Talisuna, Nataliya Podolchak, Chi K Nguyen, Silas Quaye, Annie Mwila, Ponesai Nyika

**Affiliations:** ^1^Division for Global HIV and TB, Center for Global Health, CDC; ^2^Office of the U.S. Global AIDS Coordinator and Health Diplomacy, U.S. Department of State, Washington D.C.; CDC; CDC; CDC; CDC; CDC; CDC; CDC; CDC; CDC; CDC; CDC; CDC; CDC; CDC; CDC Brazil Country Office; CDC Caribbean Regional Office; CDC Central America Regional Office; CDC Côte d'Ivoire Country Office; CDC Democratic Republic of the Congo Country Office; CDC Dominican Republic Country Office; CDC Eswatini Country Office; CDC Ethiopia Country Office; CDC Haiti Country Office; CDC India Country Office; CDC Kazakhstan Country Office; CDC Kenya Country Office; CDC Kyrgzstan Country Office; CDC Laos Country Office; CDC Lesotho Country Office; CDC Malawi Country Office; CDC Mozambique Country Office; CDC Namibia Country Office; CDC Nigeria Country Office; CDC Philippines Country Office; CDC Rwanda Country Office; CDC South Africa Country Office; CDC South Sudan Country Office; CDC Tajikistan Country Office; CDC Tanzania Country Office; CDC Thailand Country Office; CDC Uganda Country Office; CDC Ukraine Country Office; CDC Vietnam Country Office; CDC West Africa Regional Program Country Office; CDC Zambia Country Office; CDC Zimbabwe Country Office

## Abstract

**Introduction:**

In 2004, the U.S. President’s Emergency Plan for AIDS Relief (PEPFAR), with CDC as a major U.S. government implementing agency, began providing HIV antiretroviral therapy (ART) worldwide. Through suppression of HIV viral load, effective ART reduces morbidity and mortality among persons with HIV infection and prevents vertical and sexual transmission.

**Methods:**

To describe program impact, data were analyzed from all PEPFAR programs and from six countries that have conducted nationally representative Population-based HIV Impact Assessment (PHIA) surveys, including PEPFAR programmatic data on the number of persons with HIV infection receiving PEPFAR-supported ART (2004–2022), rates of viral load coverage (the proportion of eligible persons with HIV infection who received a viral load test) and viral load suppression (proportion of persons who received a viral load test with <1,000 HIV copies per mL of blood) (2015–2022), and population viral load suppression rates in six countries that had two PHIA surveys conducted during 2015–2021. To assess health system strengthening, data on workforce and laboratory systems were analyzed.

**Results:**

By September 2022, approximately 20 million persons with HIV infection in 54 countries were receiving PEPFAR-supported ART (62% CDC-supported); this number increased 300-fold from the 66,550 reported in September 2004. During 2015–2022, viral load coverage more than tripled, from 24% to 80%, and viral load suppression increased from 80% to 95%. Despite increases in viral load suppression rates and health system strengthening investments, variability exists in viral load coverage among some subpopulations (children aged <10 years, males, pregnant women, men who have sex with men [MSM], persons in prisons and other closed settings [persons in prisons], and transgender persons) and in viral load suppression among other subpopulations (pregnant and breastfeeding women, persons in prisons, and persons aged <20 years).

**Conclusions and implications for public health practice:**

Since 2004, PEPFAR has scaled up effective ART to approximately 20 million persons with HIV infection in 54 countries. To eliminate HIV as a global public health threat, achievements must be sustained and expanded to reach all subpopulations. CDC and PEPFAR remain committed to tackling HIV while strengthening public health systems and global health security.

## Introduction

The U.S. President’s Emergency Plan for AIDS Relief (PEPFAR) was announced in January 2003 and remains the largest commitment by any nation to address a single disease. PEPFAR’s core aim is to address health inequities in access to HIV services. The initial goal was to prevent 7 million infections, treat 2 million persons, and provide humane care for persons suffering from AIDS and for children orphaned by AIDS.^†^ At the time, approximately 30 million persons with HIV infection were estimated to live on the African continent, including 3 million children and adolescents aged <15 years; however, only 50,000 were receiving antiretroviral therapy (ART).^§^ Since 2004, PEPFAR has supported partner governments’ expansion of ART delivery while strengthening health systems. Through viral load suppression, effective ART reduces morbidity and mortality among persons with HIV infection (*1*); it also prevents vertical transmission from mothers with HIV infection to their infants if the mother is on ART and the HIV-exposed infant receives prophylaxis; and prevents sexual transmission when viral load is undetectable (<200 copies per mL of blood) (*2*–*5*).

PEPFAR, led and coordinated by the U.S. Department of State, uses a whole-of-government approach for global HIV/AIDS response, implemented by seven U.S. government departments and agencies, including CDC.^¶^ As the U.S. agency responsible for protecting public health, CDC couples its core area investments in public health workforce development, surveillance, and laboratory capacity with scientific and technical expertise and data-driven approaches to fight the global HIV epidemic and other threats to global health security.**

PEPFAR supports the Sustainable Development Goals and the Joint United Nations Programme on HIV/AIDS’ (UNAIDS) fast-track strategy to end the AIDS epidemic as a global threat by 2030: that 95% of persons with HIV infection know their status, that 95% of those with known status receive ART, and that 95% of those receiving ART achieve viral load suppression.^††^ Worldwide in 2021, an estimated 38.4 million persons had HIV infection; 650,000 AIDS-related deaths and 1.5 million new infections occurred.^§§^ An estimated 28.7 million persons with HIV infection were receiving ART, and among those receiving ART, an estimated 92% had suppressed viral loads. To assess PEPFAR-supported program impact and health system–strengthening investments, programmatic data from all PEPFAR programs and survey data for six countries with more than one Population-based HIV Impact Assessment (PHIA) survey were analyzed.^¶¶^

## Methods

To describe program impact, PEPFAR Monitoring, Evaluation, and Reporting*** programmatic data were analyzed by age, sex, and subpopulation (pregnant or breastfeeding women and key populations, including female sex workers, men who have sex with men (MSM), transgender persons, persons who inject drugs, and persons in prisons), and proportion of CDC contribution; analyses were stratified by fiscal year (October–September).^†††^ Before October 2018, persons with HIV infection receiving PEPFAR-supported ART were defined as persons currently receiving ART and for whom ≤90 days had elapsed after missing a scheduled ART pickup; in October 2018, this definition changed to persons currently receiving ART for whom ≤28 days had elapsed after missing a scheduled ART pickup. A proxy rate for viral load coverage was calculated as the percentage of persons with HIV infection receiving ART for ≥6 months with documented receipt of a viral load test within the previous 12 months. As an indicator of ART effectiveness, viral load suppression was defined as <1,000 HIV copies per mL of blood, and the viral load suppression rate was calculated as the number of persons with HIV infection with viral load suppression among those who received a viral load test. Using data from the PEPFAR-supported, CDC-led PHIA surveys, population viral load suppression rates by sex and age group (15–24, 25–34, 35–49, and ≥50 years) were analyzed for six countries (Eswatini, Lesotho, Malawi, Uganda, Zambia, and Zimbabwe) that completed two surveys during 2015–2021.^§§§^

PEPFAR Monitoring, Evaluation, and Reporting data were analyzed to describe health system strengthening investments. The workforce includes the number of health care workers (including lay, clinical, pharmacy, and laboratory workers) who provide HIV- or tuberculosis (TB)-related prevention, treatment, or other HIV-related services in community, clinic, or other settings. Molecular testing capacity was defined as the existence of a facility with dedicated infrastructure and staff members trained to conduct HIV early infant diagnosis, viral load, or TB molecular diagnostic testing. Laboratory continuous quality improvement enrollment was defined as participation in activities aimed at ensuring diagnostic accuracy and reliability supported by a recognized laboratory continuous quality improvement program. Accreditation was defined as achieving the highest standard of clinical laboratory quality as assessed by a nationally, regionally, or internationally recognized accrediting body. This activity was reviewed by CDC and conducted consistent with applicable federal law and CDC policy.^¶¶¶^

## Results

**FIGURE 1:**
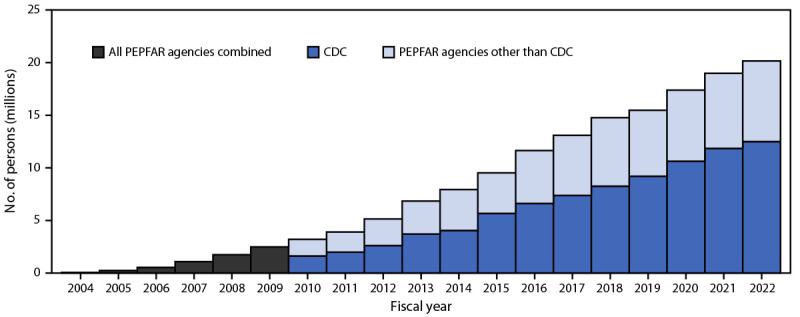
Cumulative number of persons with HIV infection receiving antiretroviral therapy supported by the U.S. President’s Emergency Plan for AIDS Relief,* by CDC and other agencies^†^ — worldwide,^§^ fiscal years 2004–2022^¶^ **Abbreviations**: ART = antiretroviral therapy; PEPFAR = U.S. President’s Emergency Plan for AIDS Relief. * Data on support provided by CDC and other PEPFAR agencies available for fiscal years 2010–2022. ^†^ PEPFAR agencies include the U.S. Agency for International Development, the U.S. Department of Health and Human Services and its agencies (CDC, Health Resources and Service Administration, and National Institutes of Health), the U.S. Department of Defense, the Peace Corps, the U.S. Department of Labor, the U.S. Department of Commerce, and the U.S. Department of the Treasury. ^§^ As of September 30, 2022: Angola, Benin, Botswana, Brazil, Burkina Faso, Burma, Burundi, Cameroon, Colombia, Cote d'Ivoire, Democratic Republic of the Congo, Dominican Republic, El Salvador, Eswatini, Ethiopia, Ghana, Guatemala, Haiti, Honduras, India, Indonesia, Jamaica, Kazakhstan, Kenya, Kyrgyzstan, Laos, Lesotho, Liberia, Malawi, Mali, Mozambique, Namibia, Nepal, Nicaragua, Nigeria, Panama, Papua New Guinea, Peru, Philippines, Rwanda, Senegal, Sierra Leone, South Africa, South Sudan, Tajikistan, Tanzania, Thailand, Togo, Trinidad and Tobago, Uganda, Ukraine, Vietnam, Zambia, and Zimbabwe. ^¶^ Fiscal years are October–September.

**FIGURE 2:**
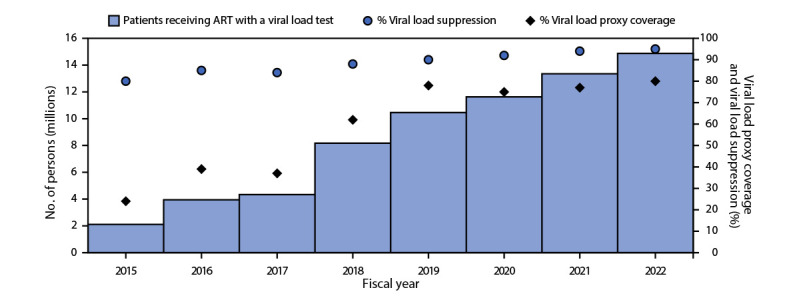
Number of persons with HIV infection receiving antiretroviral therapy supported by the U.S. President’s Emergency Plan for AIDS Relief with a viral load test,* viral load proxy coverage rate,^†^ and viral load suppression rate^§^ — worldwide,^¶^ fiscal years 2015–2022** **Abbreviations:** ART = antiretroviral therapy; PEPFAR = U.S. President’s Emergency Plan for AIDS Relief. * Viral load test result documented in the patient record or laboratory information system. ^†^ Proxy viral load coverage rate was calculated as the percentage of persons with HIV infection receiving ART for ≥6 months with documented receipt of a viral load test within the preceding 12 months. ^§^ Viral load suppression was defined as <1,000 HIV copies per mL of blood; suppression rate was calculated as the number of persons with HIV infection with viral load suppression among those who received a viral load test. ^¶^ As of September 30, 2022: Angola, Benin, Botswana, Brazil, Burkina Faso, Burma, Burundi, Cameroon, Colombia, Cote d'Ivoire, Democratic Republic of the Congo, Dominican Republic, El Salvador, Eswatini, Ethiopia, Ghana, Guatemala, Haiti, Honduras, India, Indonesia, Jamaica, Kazakhstan, Kenya, Kyrgyzstan, Laos, Lesotho, Liberia, Malawi, Mali, Mozambique, Namibia, Nepal, Nicaragua, Nigeria, Panama, Papua New Guinea, Peru, Philippines, Rwanda, Senegal, Sierra Leone, South Africa, South Sudan, Tajikistan, Tanzania, Thailand, Togo, Trinidad and Tobago, Uganda, Ukraine, Vietnam, Zambia, and Zimbabwe. ** Fiscal years are October–September.

During 2004–2022, the number of persons with HIV infection receiving PEPFAR-supported ART increased 300-fold, from 66,550 to 20,166,110, in 54 countries (Figure 1). During 2015–2022, the annual number of persons with HIV infection who received a viral load test increased 605%, from 2,109,749 to 14,875,130, and the overall viral load coverage rate increased 233%, from 24% (2,109,749 of 8,806,300 eligible persons who received a viral load test) to 80% (14,875,130 of 18,573,406) (Figure 2) (Table 1). During 2017–2022, viral load coverage rates increased to approximately 75% among women, men, and persons aged <10, 10–19, and ≥20 years; among pregnant women, viral load coverage increased 72%, from 18% to 31%. During 2020–2022, viral load coverage increased from 70% to 85% among female sex workers, from 62% to 83% among persons who inject drugs, and from 64% to 78% among MSM. Among transgender persons, viral load coverage decreased 6%, from 71% to 67%, and among persons in prisons, coverage decreased 24%, from 75% to 57%.

**TABLE 1:** Summary of programmatic data on viral load testing,* proxy viral load coverage,^†^ and viral load suppression,^§^ by age group, sex, and subpopulation — worldwide, fiscal years 2015–2022^¶^

Characteristic (program fiscal years, 2015–2022)^¶^	Baseline programmatic data**			Programmatic data from September 30, 2022			Change^§§^ in VL proxy coverage, baseline–2022, %	Change^¶¶^ in VL suppression, baseline–2022, %
	No. of eligible^††^ persons receiving VL test/total eligible persons	VL proxy coverage rate, %	VL suppression rate, %	No. of eligible^††^ persons receiving VL test/total eligible persons	VL proxy coverage rate, %	VL suppression rate, %		
**Overall**	**2,109,749/8,806,300**	**24**	**80**	**14,875,130/18,573,406**	**80**	**95**	**233**	**19**
**Age group, yrs (2017–2022)**								
<10	147,143/313,426	47	67	249,530/314,058	79	84	68	25
10–19	240,705/496,053	49	68	620,686/729,305	85	88	73	29
≥20	3,649,299/8,403,258	43	86	13,941,086/17,202,330	81	96	88	12
Unknown***	249,150/2,832,066	9	88	63,828/327,713	19	94	111	7
**Sex^†††^ (2017–2022)**								
Female	2,760,201/7,695,526	36	86	9,768,760/12,034,211	81	95	125	10
Male	1,339,638/4,029,411	33	84	5,106,370/6,539,195	78	95	136	13
**Pregnant and breastfeeding women with HIV infection^§§§^ (2017–2022)**								
Pregnant women	80,652/438,315	18	95	150,818/487,608	31	92	72	−3
Breastfeeding women	82,255	NA	85	399,082	NA	94	—	11
**Key populations (2020–2022)**								
Female sex workers	58,378/83,095	70	93	199,435/233,652	85	97	21	4
Men who have sex with men	51,317/79,983	64	94	165,352/210,926	78	97	22	3
Persons in prisons and other enclosed settings	18,605/24,821	75	93	22,836/39,805	57	93	–24	0
Persons who inject drugs	33,716/54,394	62	93	79,822/96,228	83	96	34	3
Transgender persons	2,352/3,328	71	89	7,120/10,700	67	96	−6	8

**Abbreviations:** ART = antiretroviral therapy; NA = not applicable; PEPFAR = U.S. President’s Emergency Plan for AIDS Relief; VL = viral load.

* VL test result documented in the patient record or laboratory information system.

^†^ Proxy VL coverage rate was calculated as the percentage of persons with HIV infection who received ART for ≥6 months with documented receipt of a VL test within the preceding 12 months.

^§^ VL suppression was defined as <1,000 HIV copies per mL of blood; suppression rate was calculated as the number of persons with HIV infection with VL suppression among those who received a VL test.

^¶^ Each characteristic was calculated using data from the end of the fiscal year from which they were available to the end of fiscal year 2022.

** The year of comparison for each group varied based on the quality and availability of data for analyses and is indicated in the row parentheses.

^††^ Eligible persons are those who have received ART for ≥6 months, derived from the number of persons receiving PEPFAR-supported ART during the two preceding quarters.

^§§^ Calculated as ([VL coverage 2022 − VL coverage baseline]/VL coverage baseline) * 100.

^¶¶^ Calculated as ([VL suppression 2022 − VL suppression baseline]/VL suppression baseline) * 100.

*** The value for age unknown includes 327,713 persons who received ART and were eligible for a VL test, reported in aggregate age groups only (i.e., <15 years and ≥15 years). Because of the proxy nature of the indicator, data reporting discrepancies for age group (<15 years and ≥15 years versus age disaggregates in ≤5-year age bands) might be observed.

^†††^ PEPFAR indicators are disaggregated by biologic sex (male or female), where applicable.

^§§§^ The number of breastfeeding women receiving ART is not reported in PEPFAR monitoring, evaluation, and reporting.

During 2015–2022, the viral load suppression rate among those receiving testing increased from 80% (1,691,232 persons with viral load suppression of 2,109,749 who received a viral load test) to 95% (14,146,647 of 14,875,130) (Figure 2). During 2017–2022, the viral load suppression rate increased among women, men, persons aged <10, 10–19, and ≥20 years, pregnant women, and breastfeeding women. Males, females, and those aged ≥20 years reached viral load suppression rates of ≥95% in 2022 (Table 1). By 2022, the viral load suppression rate among female sex workers, MSM, transgender persons, and persons who inject drugs reached ≥95%, but among persons in prisons, remained unchanged, at 93%.

PHIA survey results demonstrated increased population viral load suppression rates in all six assessed countries, with overall viral load suppression rates in the first and second surveys ranging from 59.2% (Zambia) to 73.1% (Eswatini) and 75.4% (Uganda) to 88.6% (Eswatini), respectively (Table 2). Across all surveys, with few exceptions, population viral load suppression rates were higher in older than in younger persons, and higher in women than in men.

**TABLE 2:** Population viral load suppression prevalence* results from Population-based HIV Impact Assessment surveys in countries supported by the U.S. President’s Emergency Plan for AIDS Relief^†^ — six African countries, 2015–2021

Country, age group, yrs	Population viral load suppression rate,%						% Change from survey 1 to survey 2		
	Survey 1			Survey 2					
	Male	Female	Total	Male	Female	Total	Male	Female	Total
**Eswatini**									
**All ages**	**67.6**	**76.0**	**73.1**	**86.1**	**90.1**	**88.6**	**18.5**	**14.1**	**15.5**
15–24	32.9	55.5	**50.6**	80.5	76.1	**77.1**	47.6	20.6	**26.5**
25–34	54.8	73.5	**68.4**	62.9	85.7	**80.4**	8.1	12.2	**12.0**
35–49	71.5	82.7	**78.5**	88.9	93.8	**91.9**	17.4	11.1	**13.4**
≥50	86.4	85.3	**85.8**	94.3	96.3	**95.3**	7.9	11.0	**9.5**
**Lesotho**									
**All ages**	**63.4**	**70.5**	**67.6**	**77.1**	**83.4**	**81.0**	**13.7**	**12.9**	**13.4**
15–24	51.3	50.9	**51.0**	61.7	65.6	**64.7**	10.4	14.7	**13.7**
25–34	46.1	64.6	**57.9**	58.7	77.6	**72.3**	12.6	13.0	**14.4**
35–49	67.5	78.3	**73.3**	78.3	87.8	**83.5**	10.8	9.5	**10.2**
≥50	84.3	80.6	**82.3**	90.7	90.7	**90.7**	6.4	10.1	**8.4**
**Malawi**									
**All ages**	**60.9**	**73.1**	**68.3**	**85.5**	**88.4**	**87.3**	**24.6**	**15.3**	**19.0**
15–24	37.2	49.7	**46.0**	75.0	73.2	**73.8**	37.8	23.5	**27.8**
25–34	48.2	70.1	**62.9**	74.0	82.6	**80.1**	25.8	12.5	**17.2**
35–49	66.0	78.5	**73.2**	87.6	92.7	**90.8**	21.6	14.2	**17.6**
≥50	73.7	81.6	**78.0**	90.9	93.0	**92.0**	17.2	11.4	**14.0**
**Uganda**									
**All ages**	**53.6**	**62.9**	**59.6**	**69.8**	**78.3**	**75.4**	**16.2**	**15.4**	**15.8**
15–24	32.5	44.9	**42.5**	43.5	57.8	**54.7**	11.0	12.9	**12.2**
25–34	38.7	57.9	**52.6**	51.9	75.0	**68.8**	13.2	17.1	**16.2**
35–49	60.1	71.4	**66.3**	75.1	84.9	**80.9**	15.0	13.5	**14.6**
≥50	65.0	79.4	**73.0**	85.4	90.2	**88.0**	20.4	10.8	**15.0**
**Zambia**									
**All ages**	**57.2**	**60.4**	**59.2**	**85.5**	**86.6**	**86.2**	**28.3**	**26.2**	**27.0**
15–24	36.7	33.6	**34.3**	70.1	71.2	**70.9**	33.4	37.6	**36.6**
25–34	36.7	56.1	**50.4**	72.6	83.4	**81.0**	35.9	27.3	**30.6**
35–49	61.8	70.8	**66.9**	87.7	89.9	**89.1**	25.9	19.1	**22.2**
≥50	79.7	73.5	**76.6**	93.0	91.7	**92.3**	13.3	18.2	**15.7**
**Zimbabwe**									
**All ages**	**54.1**	**63.8**	**59.8**	**73.0**	**79.8**	**77.3**	**18.9**	**16.0**	**17.5**
15–24	40.1	47.9	**45.3**	49.2	66.2	**60.6**	9.1	18.3	**15.3**
25–34	36.2	54.2	**48.7**	52.4	70.7	**65.7**	16.2	16.5	**17.0**
35–49	55.8	70.5	**63.9**	76.6	82.4	**80.2**	20.8	11.9	**16.3**
≥50	71.6	78.8	**75.1**	84.5	91.0	**88.1**	12.9	12.2	**13.0**

* Viral load suppression was defined as <1,000 copies per mL of blood; suppression rate was calculated as the number of persons with HIV infection with viral load suppression among those who received a viral load test.

^†^ Eswatini: survey 1 was conducted during 2016–2017, and survey 2 during 2021; Lesotho: survey 1 was conducted during 2016–2017, and survey 2 during 2020; Malawi: survey 1 was conducted during 2015–2016, and survey 2 during 2020–2021; Uganda: survey 1 was conducted during 2016–2017, and survey 2 during 2020–2021; Zambia: survey 1 was conducted during 2016, and survey 2 during 2021; and Zimbabwe: survey 1 was conducted during 2015–2016, and survey 2 during 2021.

In 2022, the PEPFAR-supported workforce included 371,760 health care workers in approximately 70,000 community, clinic, or other settings. During 2017–2022, the number of PEPFAR-supported facilities with a molecular laboratory increased by 115%, from 926 to 1,995; the number of PEPFAR-supported facilities with one or more laboratory enrolled in a continuous quality improvement program increased by 112%, from 795 to 1,687; and those that were accredited increased by 194%, from 103 to 303.

In 2010, approximately one half of persons with HIV infection receiving PEPFAR-supported ART received services through CDC implementing partners (Figure 1). By September 2022, CDC implementing partners supported 62% (12,566,736 of 20,166,110 persons with HIV infection receiving PEPFAR-supported ART) of the PEPFAR total. Among the total PEPFAR-supported workforce in 2022, 42% were supported through CDC implementing partners.

## Discussion

The cumulative program impact of PEPFAR among 54 countries reached approximately 20.2 million persons with HIV infection with lifesaving ART by September 2022, a 300-fold increase from 2004. PEPFAR-supported ART is effective, as demonstrated by program data indicating that the UNAIDS target for viral load suppression was achieved in 2022, and by PHIA survey data indicating increased viral load suppression rates at the population level (i.e., not restricted to persons with HIV infection receiving PEPFAR-supported ART). By providing effective ART, PEPFAR’s investments have helped avert new HIV infections (*6*) and have led to sustained declines in all-cause mortality.**** For example, in Uganda, the first PEPFAR-supported country, ART scale-up since 2004 has helped to avert an estimated 500,000 infections, including approximately 230,000 infections among HIV-exposed infants, and 600,000 HIV-related deaths (*7*). In Eswatini, national HIV incidence decreased by nearly one half and viral load suppression doubled during 2011–2016 (*8*).

PEPFAR program impact is founded on strengthened health systems. Improvements in laboratory capacity, including molecular testing and continuous quality improvement activities described in this report, have supported the full HIV cascade of care (*9*), including accurate HIV diagnosis, treatment, and viral load monitoring of ART effectiveness. Investments reflect PEPFAR’s commitment to local public health system strengthening for broader pandemic preparedness and response. Under PEPFAR’s current 5-year strategy,^††††^ the United States aims to eliminate the HIV/AIDS pandemic as a public health threat by 2030, while sustainably strengthening public health systems.

Through PEPFAR, CDC is at the forefront of global ART scale-up efforts. CDC receives approximately 50% of PEPFAR funding for HIV treatment and supports approximately 60% of all persons receiving ART through PEPFAR. The PEPFAR-supported CDC-led PHIA surveys have provided rigorous estimates of critical HIV indicators by age group, sex, and subnational geographic units. Other PEPFAR investments achieved through CDC have strengthened surveillance systems, such as health and laboratory information systems for patient and program monitoring, as well as HIV case reporting. The PEPFAR laboratory continuous quality improvement program (Strengthening Laboratory Management Toward Accreditation^§§§§^) has provided practical tools for resource-limited settings to improve quality management systems and prepare laboratories for accreditation^¶¶¶¶^ (*10*). CDC provides leadership in the use of multiple data sources to continually identify gaps in HIV service delivery for policy and program action (*11*,*12*).

Beyond HIV, PEPFAR investments in public health system strengthening have had additional benefits, including improving global health security. For example, during the COVID-19 pandemic, PEPFAR-supported countries demonstrated the resilience of PEPFAR investments by protecting and advancing HIV response gains (*13*,*14*), while also responding to COVID-19. In Nigeria, an ART surge in nine states supported by CDC through PEPFAR rapidly increased the total number of persons with HIV infection receiving ART by 26% (110,815) during April–September 2020 alone (*15*,*16*). PEPFAR investments have been leveraged for public health emergency response. Workforce investments have trained and deployed large numbers of health care workers not only to prevent, diagnose, and treat HIV and provide quality care for persons with HIV infection, but also to identify, track, and contain other health threats such as cholera, Ebola virus disease, and COVID-19.***** During April 2020–March 2021, a total of 109 PEPFAR-supported centralized HIV viral load and early infant diagnosis laboratories and 138 decentralized HIV and TB sites reported conducting approximately 3.4 million SARS-CoV-2 tests in 16 countries (*17*).

Despite these achievements, 10 million persons with HIV infection worldwide (in countries with and without PEPFAR support) were not receiving ART in 2021, and gaps exist among certain subpopulations. Global HIV control cannot be achieved without prioritizing health equity. For example, although overall viral load coverage rates have increased over time, rates were lower among children aged <10 years, males, pregnant women, MSM, persons in prisons, and transgender persons. Similarly, whereas overall viral load suppression rates reached the UNAIDS target of 95% of persons with HIV infection receiving ART, rates were lower among pregnant and breastfeeding women and persons in prisons, and much lower for persons aged <20 years, including children and adolescents with HIV infection. Results from PHIA surveys further highlight lower viral load suppression rates among younger age groups and among men compared with women. Stigma and discrimination remain important barriers to health equity. In sub-Saharan Africa, for example, HIV prevalence among MSM and transgender women is significantly higher than it is in the general population (*18*). Understanding the root causes including structural determinants of health for the observed differences and addressing potential factors leading to health disparities is essential to eliminate HIV as a global public health threat.

The findings in this report are subject to at least six limitations. First, indicator definitions and the systems to collect and report data have evolved over time, which might have affected data quality and results observed. Second, the countries, number of sites reporting, changes in national HIV guidelines (i.e., prevention, treatment, ART initiation criteria, recommended ART regimens, and monitoring), and the ability for persons with HIV infection to access services at any site have also evolved, which might have affected results observed. Third, misclassification of patients in certain subpopulations might have occurred if this information was not disclosed and captured by medical records. Fourth, viral load coverage analyses used aggregate program data, and as such, reported viral load proxy coverage rates could differ from actual viral load coverage rates. Fifth, because some facilities might have more than one laboratory, the number of laboratories might have been underreported. Finally, programmatic data cannot be directly compared with PHIA results, which are derived through representative sampling methods.

Since 2004, PEPFAR has scaled up ART to approximately 20 million persons with HIV infection worldwide, managing a chronic disease at an unprecedented level while strengthening public health systems through workforce, surveillance, and laboratory capacity investments. To eliminate HIV as a global public health threat, achievements in HIV services must be sustained and expanded to reach all subpopulations. PEPFAR remains committed to supporting partner governments to eliminate HIV as a global public health threat while strengthening public health systems and global health security.

**Summary**

**What is already known about this topic?**

The U.S. President’s Emergency Plan for AIDS Relief (PEPFAR) began providing HIV antiretroviral therapy (ART) worldwide in 2004. Through viral load suppression, effective ART improves health outcomes and prevents transmission.

**What is added by this report?**

By 2022, approximately 20 million persons with HIV infection in 54 countries received PEPFAR-supported ART (62% CDC-supported); this number represents an increase of 300-fold from 66,550 in 2004. During 2015–2022, viral load suppression rates increased from 80% to 95% among those who received testing.

**What are the implications for public health practice?**

To eliminate HIV as a global public health threat, achievements must be sustained and expanded to reach all subpopulations. PEPFAR remains committed to tackling HIV while strengthening public health systems and global health security.

## Footnotes

* These authors contributed equally to this report.

†
https://www.state.gov/pepfar/

§
https://www.washingtonpost.com/wp-srv/onpolitics/transcripts/bushtext_012803.html

¶
https://www.state.gov/about-us-pepfar/

**
https://www.cdc.gov/globalhivtb/index.html

†† https://www.unaids.org/en/resources/documents/2022/in-danger-global-aids-update; https://www.unaids.org/en/resources/fact-sheet

§§
https://www.unaids.org/en/resources/campaigns/World-AIDS-Day-Report-2014

¶¶
https://www.cdc.gov/globalhivtb/what-we-do/phia/phia.html

***
https://www.state.gov/wp-content/uploads/2021/09/FY22-MER-2.6-Indicator-Reference-Guide.pdf

††† For persons with HIV infection receiving PEPFAR-supported ART, overall data were available for 2004–2022; data by agency were analyzed for 2010–2022; and data by age, sex, and key population were analyzed for 2022. For viral load, overall data were available for 2015–2022; data by age, sex, and among pregnant or breastfeeding women were available for 2017–2022; data on key populations were available for 2020–2022; and data were not available to calculate viral load proxy coverage among breastfeeding women. Data on human resources to support HIV and TB services were available for 2022. Data on laboratory capacity were available for 2017–2022. PEPFAR indicators are disaggregated by biologic sex (male or female), where applicable.

§§§ Population viral load suppression rate was calculated as the number of persons with HIV infection with viral load suppression (<1,000 HIV copies per mL of blood) among all persons identified with HIV infection. PHIA survey data sets are available for public download from the PHIA Project team portal (https://phia-data.icap.columbia.edu/datasets). Eswatini: survey 1 was conducted during 2016–2017, and survey 2 during 2021; Lesotho: survey 1 was conducted during 2016–2017, and survey 2 during 2020; Malawi: survey 1 was conducted during 2015–2016, and survey 2 during 2020–2021; Uganda: survey 1 was conducted during 2016–2017, and survey 2 during 2020–2021; Zambia: survey 1 was conducted during 2016, and survey 2 during 2021; and Zimbabwe: survey 1 was conducted during 2015–2016, and survey 2 during 2021.

¶¶¶ 45 C.F.R. part 46.102(l)(2), 21 C.F.R. part 56; 42 U.S.C. Sect. 241(d); 5 U.S.C. Sect. 552a; 44 U.S.C. Sect. 3501 et seq.

**** https://www.kff.org/global-health-policy/issue-brief/assessing-pepfars-impact-analysis-of-mortality-in-pepfar-countries/; https://www.state.gov/pepfar/

††††
https://www.state.gov/pepfar-five-year-strategy-2022/

§§§§
https://www.slmta.org

¶¶¶¶
https://www.cdc.gov/globalhealth/stories/2022/cdc-laboratory-program-prepares-countries-COVID-19.html

*****
https://www.state.gov/wp-content/uploads/2021/06/06.23.21-PEPFAR-Technical-Guidance-During-COVID-final.pdf
